# Arsenic in Wall-Paper

**Published:** 1875-04

**Authors:** 


### ARSENIC IN WALL-PAPER.

This poison is largely employed as a coloring in green paper. We tested a beautiful sample the other day, and found in a square foot, thirty-seven and a-half grains of pure arsenic. The least rubbing set free a cloud of green dust, which was rank poison to inhale. A few weeks since a physician was asked to see, in consultation, a brother medical man who was dangerously ill of erysipelas of the face and scalp. He had only just removed into a new house. On entering his consulting-room, the new comer was struck with the bright green of the wall-paper, and asked to have a piece supplied to him. After some time this was done, when he found, on examining it, that it was, as he anticipated, arsenical. It is not asserted that the newly-hung wall-paper had anything to do with the attack; but the simple suggestion is offered, that, as the methods for discovering arsenic are so simple and so accurate, medical men should always take care to guard themselves, at least; and, without much trouble, they may guard their patients also against the risk of injury from this source. If, on burning some of the suspected paper in the center of the room, so that the fumes can be inhaled, the odor of garlic or onions is apparent, arsenic is present, and the paper should be condemned.

---

Persons always busy in things laudable or praiseworthy, and who go cheerfully to their daily tasks, are the least disturbed by the fluctuations of business; and, at night, sleep with perfect composure.

### SEEING STARS.

If a man falls so as to strike his head violently on the ice or on the pavement, or if he gets a blow over his eye, he is said to "see stars." The cause of this curious phenomenon is found in a peculiarity of the optic nerve. The function of that nerve is to convey to the brain the impression of light. It recognizes nothing in the world but light. It is susceptible to no other impression. Or, if acted upon by any other agent, it communicates to the brain the intelligence of the presence of that agent by sending along its fibers, flashes of light only. Irritate this nerve with a probe or other instrument, and it conveys no sensation of pain, but simply that of luminous sparks. The pain of the blow on the eye, or the fall on the head, is realized through the nerves of general sensation; but, unsusceptible to pain or any other feeling, the optic nerve sends to the brain its report of the shock by flashes, sparks, and "stars." The young doctor who died recently after falling upon his umbrella and puncturing his eye and brain with one of its steel ribs, complained of intense light in the eye forever darkened.

---

Both young men and young women should be taught that it is no sacrifice of respectability to begin married life with bare comforts and few luxuries, where pecuniary ability will warrant no more; and society should guaranty them against all loss of social standing by so doing.

---

Remember many men have done well in very small shops.

